# Epidemiological and clinical profile of ischemic, hemorrhagic, and mixed strokes in Sikasso, Mali: a prospective cross-sectional study

**DOI:** 10.11604/pamj.2026.53.6.49624

**Published:** 2026-01-07

**Authors:** Abdoulaye Kissima Traoré, Kadidiatou Diarra, Ousmane Haidara

**Affiliations:** 1 Department of Cardiology, Sikasso Hospital, Sikasso, Mali,; 2 Cheikh Anta Diop University, Dakar, Senegal,; 3 Institute for Health and Development, Cheikh Anta Diop University, Dakar, Senegal

**Keywords:** Stroke, clinical profiles, hypertension, multiple correspondence analysis, Mali

## Abstract

**Introduction:**

in sub-Saharan Africa, stroke places a substantial burden on health systems and populations. Clinical and epidemiological features vary by subtype, ischemic, hemorrhagic, and mixed, but data specific to Mali are limited. This study characterises, in Sikasso, the profiles linked to each stroke type.

**Methods:**

from November 2021 to October 2022, a prospective cross-sectional study included 135 CT-confirmed stroke admissions. Socio-demographic, clinical, and biological information was collected at presentation. We used descriptive and bivariate statistics and performed multiple correspondence analysis (MCA) to delineate profiles for ischemic, hemorrhagic, and mixed strokes.

**Results:**

patients had a mean age of 60.4 ± 12.6 years; subtype distribution was ischemic 74.8%, hemorrhagic 21.5%, mixed 3.7%. Severe hypertension was predominant in the hemorrhagic group, whereas hyperglycemia was more common in patients with ischemic or mixed strokes. The MCA identified distinct profiles. Hemorrhagic stroke: cardiovascular profile marked by grade 2-3 hypertension and left ventricular hypertrophy. Mixed stroke: metabolic and cardiac profiles associated with ischemic cardiomyopathy and living in the suburbs. Ischemic stroke: heterogeneous profile with no dominant modality.

**Conclusion:**

this study highlights the different clinical profiles depending on the stroke type, reinforcing the importance of tailored care and targeted prevention strategies in Sikasso. The results underscore the central role of severe hypertension in hemorrhagic strokes and hyperglycemia in ischemic and mixed strokes.

## Introduction

Across sub-Saharan Africa, stroke poses a major public health challenge, standing among the top drivers of premature adult mortality and disability [1,2]. The World Stroke Organization notes that LMICs shoulder over four-fifths of the global stroke burden, with Africa's incidence still climbing [1]. These contexts absorb more than two-thirds of the total burden, with annual costs near 890 billion US dollars and projections indicating further increases by 2050 [3]. The diversity of epidemiological and clinical profiles according to stroke type (ischemic, hemorrhagic, or mixed) is well recognised, with ischemic stroke predominating in most series; however, data remain heterogeneous and are often limited to hospital cohorts in Africa. This heterogeneity and gaps in structured surveillance complicate inter-study comparisons and service planning [2,4,5].

In Mali, recent publications have mainly shown single-centre data (primarily from Bamako) confirming the burden of stroke and the challenges of accessing imaging and specialised care (prehospital pathways, costs borne by patients), illustrating the need for more detailed analyses in inland regions such as Sikasso [6,7]. Socio-demographic factors (age, sex, and place of residence), clinical factors (high blood pressure, diabetes, and cardiovascular history), biological factors (e.g., hyperglycemia), and factors related to access to care (prehospital delays and rapid imaging) influence the occurrence, type, and prognosis of stroke. In sub-Saharan Africa, these determinants are associated with a high risk of early in-hospital mortality, particularly after hemorrhagic stroke [2,5,8-10].

In this context, systematically describing patient profiles according to stroke type is essential for adapting prevention and healthcare strategies in resource-constrained regions. This study aimed to characterise variations in the socio-demographic, clinical, and biological characteristics of patients hospitalised for stroke according to type (ischemic, hemorrhagic, or mixed) using a multidimensional approach based on multiple correspondence analysis (MCA) to map profiles and inform treatment priorities [5].

## Methods

**Design and study setting:** we conducted a prospective cross-sectional investigation at Sikasso Regional Hospital (Mali) between November 2021 and October 2022. Methods and reporting were aligned with STROBE guidance (checklist available in Supplementary material). The hospital functions as the region's principal stroke referral center.

**Study population:** all patients hospitalised for stroke confirmed by brain scan were included, regardless of age or sex. Patients with diagnoses other than stroke or without confirmation by imaging were excluded.

**Sampling:** sample size estimation followed the single-proportion formula described by Schwartz *et al*. [11]: n = Z^2^.p(1−p)/m^2^. Let n denote the required sample, Z the standard normal deviate for a 95% confidence level (1.96), p the anticipated prevalence of delayed treatment, and m the allowable absolute error. We chose p = 0.50 to optimise sample size. Under 8.5% absolute error and 95% confidence, n was ≈133; we set the sample at 135.

**Data collection:** data were collected using standardised forms completed by trained staff. The variables studied were as follows:

***Socio-demographic variables:*** age, sex, place of residence, occupation.

***Behavioural variables:*** smoking, alcohol consumption, and physical activity.

***Clinical variables:*** medical history, blood pressure, BMI, ECG, and echocardiography.

***Biological variables:*** blood sugar, lipid profile, and other blood tests.

***Dependent variable:*** type of stroke (ischemic, hemorrhagic, mixed).

It is important to note that this analysis is based on the same hospital database used in previous studies, but it focuses specifically on the distinct epidemiological and clinical profiles of each type of stroke.

**Data processing and statistical analysis:** analyses were run in R 4.4.2. Categorical data are reported as counts, proportions, and 95% CIs. Continuous data are summarised as mean (SD) or median [IQR], according to distribution. Associations with stroke subtype were examined using χ^2^ or Fisher's exact tests, as appropriate. Multiple correspondence analysis (MCA) was applied to delineate subtype-specific profiles.

**Ethical considerations:** the study was reviewed and approved by the management of Sikasso Hospital. Written consent was secured from participants or from a legally authorised representative when necessary. Data were anonymised and handled per the Declaration of Helsinki and applicable regulations.

## Results

### Descriptive results

***General characteristics of the study population:*** of the 135 enrolled patients, the mean age was 60.4 ± 12.6 years, and the median age was 62 years (27-88). The most represented age group was 40-65 years (50.4%). Most patients (75.7%) lived on the outskirts of Sikasso. The male-to-female ratio was 1.33. The most common occupations were housewives (42.2%) and farming (35.6%) (Table 1). Hypertension was reported in 30.4% of participants, including 11.1% with severe HTN (grade 3). Hyperglycemia was observed in 24.4% of patients, and left ventricular hypertrophy was observed in 30.4% of patients on ECG (Table 2).

**Table 1:** socio-demographic, economic, behavioural, and access profile

Variables	Categories	Absolute frequency (n)	Relative frequency (%)	95% CI
**Socio-demographic and economic characteristics**				
Age (n =135)	[27-40[years	6	4.4	[1.0 - 7.9]
	[40-65[years	68	50.4	[41.9 - 58.8]
	65 and over	61	45.2	[36.8 - 53.6]
Sex (n =135)	Male	77	57.0	[48.7 - 65.4]
	Female	58	43.0	[34.6 - 51.3]
Residence (n =135)	Hamdallaye	6	4.4	[1.0 - 7.9]
	Wayerma	10	7.4	[3.0 - 11.8]
	Sanoubougou	6	4.4	[1.0 - 7.9]
	Lafiabougou	5	3.7	[0.5 - 6.9]
	Mancourani	1	0.7	[0.01 - 4.0]
	Other neighborhoods of Sikasso	5	3.7	[0.5 - 6.9]
	Other localities of Sikasso	102	75.7	[68.3 - 82.8]
Profession (n =135)	Homemaker	57	42.2	[33.9 - 50.6]
	Farmer	48	35.6	[27.5 - 43.6]
	Shopkeeper	8	5.9	[1.9 - 9.9]
	Nurse	3	2.2	[0.4 - 6.3]
	Other*	19	14.1	[8.2 - 19.9]
**Behaviour-related characteristics**				
History of cardiovascular risk factors (CVRF) (n =135)	High Blood Pressure	41	30.4	[22.6 - 38.1]
	More than one cardiovascular risk factor history	72	53.3	[44.9 - 61.7]
	Other-	10	7.4	[3.0 - 11.8]
	Unknown	12	8.9	[4.1 - 13.7]

Note: Other neighbourhoods of Sikasso include Bougoula-Ville, Babemba, and Lamine Bambara. Other localities of Sikasso include Kadiolo, Koutiala, Kolondiéba, Yorosso, Kignan, Niéna, Loulouni, Fourou, Kaboila/Kaboila 2, Zégoua, Zangaradougou, Sirakoro, Siramana, Banconi, Bamako, RCI, San, Bla, Namp. Other* included Blacksmith, Driver, Retired driver, Mechanic, Painter, Tailor, Photographer, Labourer, Vendor, Repair technician, Transport operator, Imam, Marabout, and Retired. Other CVRFs included tobacco use, physical inactivity (sedentary), obesity, diabetes, and heart failure.

**Table 2:** clinical and biological characteristics

Variables	Categories	Absolute frequency (n)	Relative frequency (%)	95% CI
Body mass index (n =135)	Underweight	2	1.5	[0.2 - 5.2]
	Normal	93	68.9	[61.1 - 76.7]
	Overweight	26	19.3	[12.6 - 25.9]
	Obese	14	10.3	[5.2 - 15.5]
Hypertension grade (n=135)	Normal	40	29.6	[21.9 - 37.3]
	Grade 1 hypertension	41	30.4	[22.6 - 38.1]
	Grade 2 hypertension	39	28.9	[21.2 - 36.5]
	Grade 3 hypertension	15	11.1	[5.8 - 16.4]
Blood glucose (n=135)	Hypoglycemia	1	0.7	[0.01 - 4.0]
	Normal	101	74.8	[67.5 - 82.1]
	Hyperglycemia	33	24.5	[17.2 - 31.7]
Lipid profile (n=135)	Normal	26	19.3	[12.6 - 25.9]
	Anormal	7	5.2	[1.4 - 8.9]
	Not done	102	75.5	[67.8 - 82.5]
Echocardiography (n=135)	Normal	2	1.5	[0.2 - 5.2]
	Dilated cardiomyopathy with reduced LVEF	3	2.2	[0.4 - 6.3]
	Hypertensive cardiomyopathy	1	0.7	[0.01 - 4.0]
	Ischemic cardiomyopathy with reduced LVEF	1	0.7	[0.01 - 4.0]
	Others*	2	1.5	[0.2 - 5.2]
	Not done	126	93.4	[89.1 - 97.5]
ECG (n=135)	Normal	28	20.7	[13.9 - 27.6]
	Hypertrophy	41	30.4	[22.6 - 38.1]
	Sinus bradycardia	11	8.1	[3.5 - 12.8]
	Sinus tachycardia	20	14.8	[8.8 - 20.8]
	Extrasystoles	10	7.4	[3.0 - 11.8]
	Atrial fibrillation	4	3.0	[0.4 - 5.8]
	Others+	21	15.6	[9.4 - 21.7]

**Note:** LVEF = Left Ventricular Ejection Fraction. Others*: patterns not fitting the main categories, for example, mixed/uncertain cardiomyopathy phenotype (hypertrophic vs. hypertensive), DCM with reduced LVEF without a specified mechanism. Others+: unspecified conduction defects (1^st^-degree AV block, fascicular block, unspecified bundle branch block), axis deviation (right axis), repolarisation abnormalities (T-wave changes), Q-wave sequelae (QS pattern), atypical sinus rhythms (sinus arrhythmia/RSR), and generic “TDR” notes.

**Figure 1:**
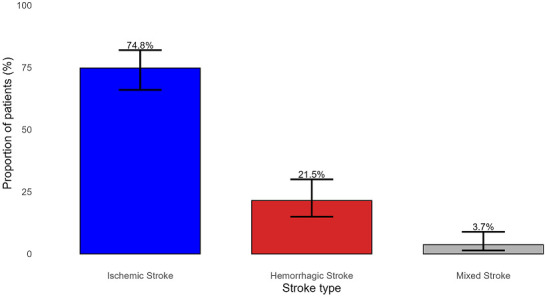
prevalence of stroke types (95% CIs)

**Distribution of stroke types:** ischemic stroke: 74.8% (95% CI: 67.5-82.1); hemorrhagic stroke: 21.5% (95% CI: 14.6-28.4) and mixed stroke: 3.7% (95% CI: 0.5-6.9) (Figure 1).

### Analytical results

***Bivariate analysis***

**Figure 2:**
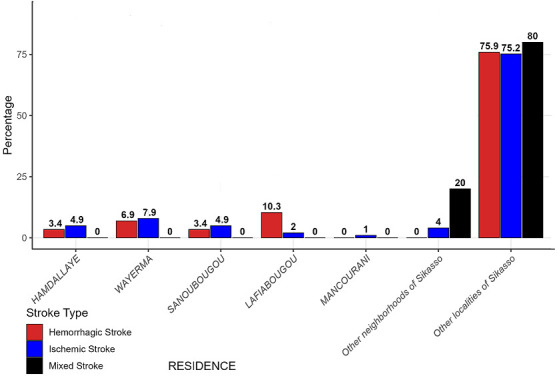
stroke type prevalence by place of residence

**Residence:** in urban areas (Lafiabougou), hemorrhagic stroke accounted for 10.3% of cases, whereas mixed stroke accounted for 80% in outlying areas (Figure 2).

**Cardiovascular risk factors (CVRF):** significant association (p = 0.01). Hypertension was more common in ischemic strokes (25.7%), especially in hemorrhagic strokes (51.7%). No cases were observed in the mixed group.

**Cumulative CVRF:** predominant in ischemic (57.4%) and mixed (80.0%) strokes compared with 34.5% in hemorrhagic strokes (Table 3).

**Table 3:** socio-demographic, economic, behavioural, and access factors linked to stroke type

Variables	Categories		Stroke type		p-value^2^
		Ischemic Stroke N = 101^1^	Hemorrhagic Stroke N = 29^1^	Mixed Stroke N = 5^1^	
**Socio-demographic and economic characteristics**					
Age					0.12
	[27-40[years	3 (3.0%)	3 (10.3%)	0 (0.0%)	-
	[40-65[years	47 (46.5%)	18 (62.1%)	3 (60.0%)	-
	65 and over	51 (50.5%)	8 (27.6%)	2 (40.0%)	-
Sex					0.2
	Male	62 (61.4%)	13 (44.8%)	2 (40.0%)	-
	Female	39 (38.6%)	16 (55.2%)	3 (60.0%)	-
Profession					0.7
	Homemaker	38 (37.6%)	16 (55.2%)	3 (60.0%)	-
	Farmer	39 (38.6%)	7 (24.1%)	2 (40.0%)	-
	Shopkeeper	6 (5.9%)	2 (6.9%)	0 (0.0%)	-
	Nurse	3 (3.0%)	0 (0.0%)	0 (0.0%)	-
	Other*	15 (14.9%)	4 (13.8%)	0 (0.0%)	
**Behavior-related characteristics**					
History of cardiovascular risk factors (CVRF)					0.01
	High blood pressure	26 (25.7%)	15 (51.7%)	0 (0.0%)	-
	More than one cardiovascular risk factor history	58 (57.4%)	10 (34.5%)	4 (80.0%)	-
	Other	10 (9.9%)	0 (0.0%)	0 (0.0%)	-
	Unknown	7 (6.9%)	4 (13.8%)	1 (20.0%)	-

**Note:** Other neighbourhoods of Sikasso include Bougoula-Ville, Babemba, and Lamine Bambara. Other localities of Sikasso include Kadiolo, Koutiala, Kolondiéba, Yorosso, Kignan, Niéna, Loulouni, Fourou, Kaboila/Kaboila 2, Zégoua, Zangaradougou, Sirakoro, Siramana, Banconi, Bamako, RCI, San, Bla, Namp. Other* included Blacksmith, Driver, Retired driver, Mechanic, Painter, Tailor, Photographer, Labourer, Vendor, Repair technician, Transport operator, Imam, Marabout, and Retired. Other CVRFs included tobacco use, physical inactivity (sedentary), obesity, diabetes, and heart failure.

**Blood pressure:** hemorrhagic strokes were characterised by severe hypertension (grades 2-3), while mixed strokes had normal BP in 80% of cases.

**Blood glucose:** hyperglycemia was observed in 27.7% of ischemic strokes, 40.0% of mixed strokes, but only 10.3% of hemorrhagic strokes (Table 4).

**Table 4:** clinical and biological factors linked to stroke type

Variables	Categories		Stroke type		p-value^2^
		Ischemic Stroke N = 101^1^	Hemorrhagic Stroke N = 29^1^	Mixed Stroke N = 5^1^	
Body mass index					0.08
	Underweight	1 (1.0%)	1 (3.4%)	0 (0.0%)	-
	Normal	67 (66.3%)	24 (82.8%)	2 (40.0%)	-
	Overweight	20 (19.8%)	4 (13.8%)	2 (40.0%)	-
	Obese	13 (12.9%)	0 (0.0%)	1 (20.0%)	-
Hypertension grade					0.003
	Normal	34 (33.7%)	2 (6.9%)	4 (80.0%)	-
	Grade 1 hypertension	32 (31.7%)	9 (31.0%)	0 (0.0%)	-
	Grade 2 hypertension	27 (26.7%)	11 (37.9%)	1 (20.0%)	-
	Grade 3 hypertension	8 (7.9%)	7 (24.1%)	0 (0.0%)	-
Blood glucose					0.006
	Hypoglycemia	0 (0.0%)	0 (0.0%)	1 (20.0%)	-
	Normal	73 (72.3%)	26(89.7%)	2 (40.0%)	-
	Hyperglycemia	28 (27.7%)	3 (10.3%)	2 (40.0%)	-
Lipid profile					>0.9
	Normal	19 (18.8%)	6 (20.7%)	1 (20.0%)	-
	Anormal	6 (5.9%)	1.00 (3.4%)	0 (0.0%)	-
	Not done	76 (75.3%)	22 (75.9%)	4 (80.0%)	-
Echocardiography					0.9
	Normal	2 (2.0%)	0 (0.0%)	0 (0.0%)	-
	Dilated cardiomyopathy with reduced LVEF	3 (3.0%)	0 (0.0%)	0 (0.0%)	-
	Hypertensive cardiomyopathy	1 (1.0%)	0 (0.0%)	0 (0.0%)	-
	Ischemic cardiomyopathy with reduced LVEF	1 (1.0%)	0 (0.0%)	0 (0.0%)	-
	Others*	1 (1.0%)	1 (3.4%)	0 (0.0%)	-
	Not done	93 (92.1%)	28 (96.6%)	5 (100%)	-
ECG					0.8
	Normal	18 (17.8%)	8 (27.6%)	2 (40.0%)	-
	Hypertrophy	29 (28.7%)	11 (37.9%)	1 (20.0%)	-
	Sinus bradycardia	9 (8.9%)	2 (6.9%)	0 (0.0%)	-
	Sinus tachycardia	16 (15.8%)	3 (10.3%)	1 (20.0%)	-
	Extrasystoles	8 (7.9%)	1 (3.4%)	1 (20.0%)	-
	Atrial fibrillation	4 (4.0%)	0 (0.0%)	0 (0.0%)	-
	Others+	17 (16.8%)	4 (13.8%)	0 (0.0%)	-

**Note:** LVEF = Left Ventricular Ejection Fraction. Others*: patterns not fitting the main categories, for example, mixed/uncertain cardiomyopathy phenotype (hypertrophic vs. hypertensive), DCM with reduced LVEF without a specified mechanism. Others+: unspecified conduction defects (1^st^-degree AV block, fascicular block, unspecified bundle branch block), axis deviation (right axis), repolarisation abnormalities (T-wave changes), Q-wave sequelae (QS pattern), atypical sinus rhythms (sinus arrhythmia/RSR), and generic ”TDR” notes.

***Multivariate analysis (MCA)***

MCA revealed three distinct profiles:

**Hemorrhagic stroke:** cardiovascular profile dominated by severe hypertension and left ventricular hypertrophy.

**Mixed stroke:** cardiometabolic profile marked by ischemic cardiomyopathy and hyperglycemia associated with peripheral residence.

**Figure 3:**
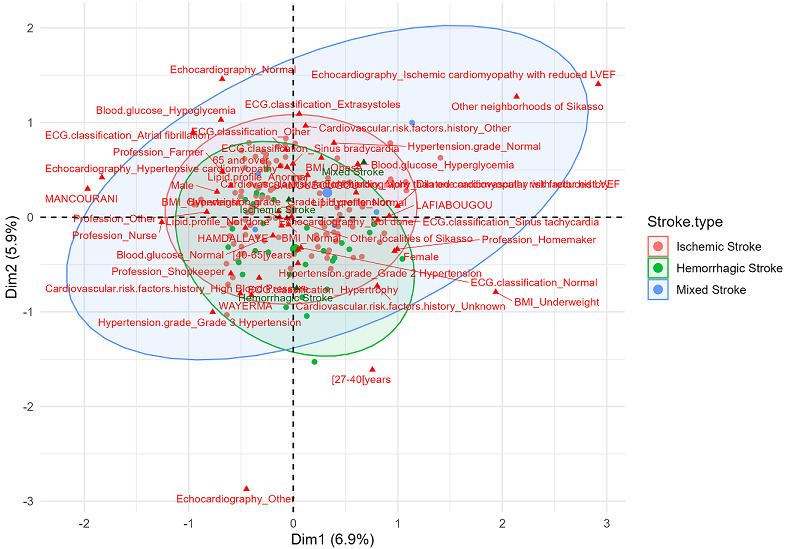
MCA of profiles associated with stroke types

**Ischemic stroke:** heterogeneous profile with no dominant modality, reflecting the diversity of contributing factors (Figure 3).

## Discussion

**Statement of key findings:** this study highlights the different clinical profiles depending on the type of stroke in the Sikasso population. Hemorrhagic stroke is strongly linked to severe hypertension and ventricular hypertrophy, whereas ischemic stroke is more frequently accompanied by hyperglycemia. Mixed stroke, although rare, presents an atypical profile that combines ischemic cardiomyopathy and peripheral habitat.

**Interpretation:** in Sikasso, hemorrhagic stroke clustered with severe hypertension and cardiac hypertrophy; ischemic stroke often coincided with hyperglycemia; mixed cases were rare and cardiometabolic. These associations are non-causal and chiefly relevant to similar settings due to the single-site design and small numbers, especially for mixed cases. They nonetheless support clear priorities: tighter BP control, routine glucose assessment, and improved access for peripheral populations.

**Comparison with other studies:** our findings are consistent with the African and international literature, which highlights the following:

The central role of high blood pressure (HBP), particularly when severe or poorly controlled, in the pathophysiology of hemorrhagic strokes (intracerebral haemorrhage, ICH). Recent review articles have confirmed that AH remains the major modifiable risk factor for ICH, with a particularly marked effect across resource-constrained settings [12-14].

A frequent connection between metabolic disorders, particularly acute hyperglycemia and diabetes, with ischemic strokes and their poor prognosis. Hyperglycemia at the onset of ischemic stroke is associated with more extensive damage, poor functional outcomes, and increased mortality; these associations persist even in patients treated with IV thrombolysis [15-17].

The difficulty of characterising “mixed” strokes (concomitant or successive ischemic and hemorrhagic forms) is still poorly described. Published cases suggest overall vascular vulnerability (e.g., microangiopathy, dissections, and systemic diseases) and highlight the rarity of these presentations, as well as the lack of specific recommendations beyond the principles of managing ischemic stroke complicated by haemorrhage or ICH with associated ischemic lesions [18,19].

More broadly, the overall stylised facts indicate a relative preponderance of ischemic strokes and a higher proportion of ICH in resource-limited settings, which is consistent with risk profiles (uncontrolled hypertension and diabetes) and constraints on access to specialised care in Africa [2,3].

**Strengths of the study:** the first systematic description of stroke profiles in Sikasso and the use of MCA allow for an innovative multidimensional approach.

**Limitations:** the monocentric design constrains how widely the results can be applied. The study had a modest sample size, especially for mixed strokes. Functional imaging data are not available, reducing diagnostic accuracy.

**Implications of the study:** Identifying specific profiles opens up opportunities to: strengthen targeted prevention (control of severe hypertension, diabetes screening); improve risk stratification in hospitals and tailor awareness campaigns for rural and peripheral areas.

**Future research:** multicenter studies incorporating socio-economic variables and qualitative approaches are needed to refine these profiles and to develop appropriate care pathways.

## Conclusion

This study highlights different stroke profiles in Sikasso: hemorrhagic stroke dominated by severe hypertension and cardiac damage, ischemic stroke associated with hyperglycemia, and “mixed” stroke characterised by a cardiometabolic phenotype common in peripheral patients, which sheds light on targeted and realistic prevention and management pathways. MCA has made it possible to map these phenotypes in an integrated manner (socio-demographic, clinical, biological, access), providing an operational tool for segmenting risk, prioritizing interventions (intensive blood pressure control, glycemic protocols, fast-track pathways for remote patients), and optimizing resource allocation (CT scan in ≤30 min, “stroke-unit lite,” and transfers). By strengthening continuity of care (early rehabilitation, secondary prevention) and reducing financial and geographical barriers, these results can improve the timeliness, quality of care, and functional outcomes. Multicenter validations and pragmatic evaluations (e.g., prehospital bundles + rapid CT scans + adapted stroke units) are now warranted to confirm the stability of the profiles and document their impact on morbidity and mortality in the Malian context.

### What is known about this topic

- Stroke is a common neurological emergency in sub-Saharan Africa;
- Severe hypertension is a key driver of hemorrhagic stroke;
- Hyperglycemia frequently accompanies ischemic stroke.

### What this study adds

- First description of stroke profiles in Sikasso (Mali);
- Identification of atypical profiles for mixed strokes;
- Methodological contribution of MCA to identify multidimensional profiles useful for prevention and management.
